# HIF-1-Dependent Reprogramming of Glucose Metabolic Pathway of Cancer Cells and Its Therapeutic Significance

**DOI:** 10.3390/ijms20020238

**Published:** 2019-01-09

**Authors:** Ayako Nagao, Minoru Kobayashi, Sho Koyasu, Christalle C. T. Chow, Hiroshi Harada

**Affiliations:** 1 Laboratory of Cancer Cell Biology, Graduate School of Biostudies, Kyoto University, Yoshida Konoe-cho, Sakyo-ku, Kyoto 606-8501, Japan; nagao.ayako.56r@st.kyoto-u.ac.jp (A.N.); kobayashi.minoru.4m@kyoto-u.ac.jp (M.K.); sho@kuhp.kyoto-u.ac.jp (S.K.); chow.tung.85r@st.kyoto-u.ac.jp (C.C.T.C.); 2 Research Center for Advanced Science and Technology, The University of Tokyo, 4-6-1 Komaba, Meguro-ku, Tokyo 153-8904, Japan

**Keywords:** hypoxia, HIF-1, glucose metabolism, cancer

## Abstract

Normal cells produce adenosine 5′-triphosphate (ATP) mainly through mitochondrial oxidative phosphorylation (OXPHOS) when oxygen is available. Most cancer cells, on the other hand, are known to produce energy predominantly through accelerated glycolysis, followed by lactic acid fermentation even under normoxic conditions. This metabolic phenomenon, known as aerobic glycolysis or the Warburg effect, is less efficient compared with OXPHOS, from the viewpoint of the amount of ATP produced from one molecule of glucose. However, it and its accompanying pathway, the pentose phosphate pathway (PPP), have been reported to provide advantages for cancer cells by producing various metabolites essential for proliferation, malignant progression, and chemo/radioresistance. Here, focusing on a master transcriptional regulator of adaptive responses to hypoxia, the hypoxia-inducible factor 1 (HIF-1), we review the accumulated knowledge on the molecular basis and functions of the Warburg effect and its accompanying pathways. In addition, we summarize our own findings revealing that a novel HIF-1-activating factor enhances the antioxidant capacity and resultant radioresistance of cancer cells though reprogramming of the glucose metabolic pathway.

## 1. Introduction

Cells produce 2, 2, and 34 molecules of adenosine 5′-triphosphate (ATP) from one molecule of glucose through glycolysis, the tricarboxylic acid (TCA) cycle (also known as the Krebs cycle or citric acid cycle), and the electron transport chain (ETC), respectively. The ETC requires molecular oxygen as the terminal acceptor of electrons for its own activity. Therefore, the TCA cycle, which does not directly use oxygen but needs the oxidized form of nicotinamide adenine dinucleotide (NAD+) supplied from the ETC, is also dependent on oxygen. In contrast to these mitochondrial pathways, glycolysis never requires oxygen, which leads to a simple hypothetical model whereby cancer cells in malignant solid tumors produce ATP mainly through mitochondrial pathways under normoxic conditions, but use glycolysis under hypoxic conditions. However, accumulated evidence has demonstrated that many cancer cells produce ATP predominantly through accelerated glycolysis followed by lactic acid fermentation, even under normoxic conditions [1,2]. The molecular basis and functions of this unique metabolic property, which is designated as the Warburg effect, have been longstanding mysteries to be solved in the research field of tumor biology.

The mysteries were solved to some extent when one of the transcription factors responsible for physiological and pathophysiological responses to hypoxia, hypoxia-inducible factor 1 (HIF-1), was cloned in the 1990s. HIF-1 was first identified as a factor inducing the expression of *erythropoietin* (*EPO*) gene for hematopoiesis [3,4], but is now recognized as a master transcription factor affecting the adaptive response to hypoxia because of its function in the induction of hundreds of genes related to angiogenesis and the reprogramming of energy metabolism [5,6,7,8]. As many cancer cells are exposed to hypoxic environments during malignant tumor growth, the metabolic reprogramming from OXPHOS to accelerated glycolysis used to be recognized as one aspect of cancer cells’ adaptive response to hypoxia. However, genetic alterations that potentially activate HIF-1 even under normoxic conditions have been repeatedly identified in cancer cells, suggesting the association of HIF-1-mediated mechanisms underlying the metabolic reprogramming with the Warburg effect [8,9,10,11]. Here, we review the latest knowledge on the mechanism of action and function of HIF-1 in the Warburg effect and the significance of both the Warburg effect and its associated pathways in the induction of antioxidant capacity and radioresistance in cancer cells [12,13,14,15].

## 2. Regulation of HIF-1 Activity: From Canonical to Non-Canonical Mechanisms

### 2.1. Canonical Mechanism

HIF-1 is a heterodimeric transcription factor consisting of the HIF-1α and HIF-1β/Aryl hydrocarbon receptor nuclear translocator (Arnt) [16,17]. HIF-1α functions as the main regulatory subunit of HIF-1 activity; on the other hand, HIF-1β is recognized to be less important in terms of the regulation of HIF-1 activity because its mRNA and protein are maintained at constant levels.

**Figure 1:**
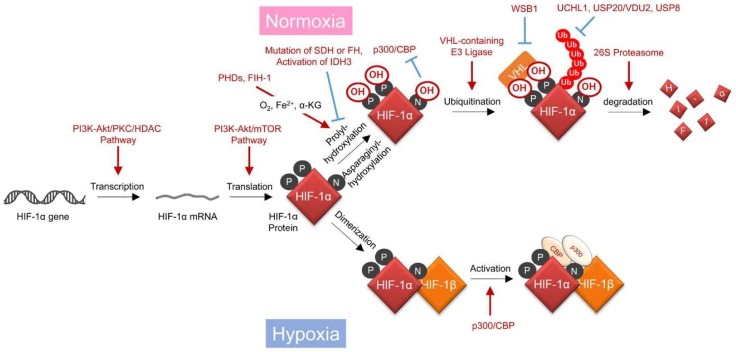
Oxygen- and hydroxylase-dependent mechanisms regulating hypoxia-inducible factor 1 (HIF-1) activity. PI3K: phosphoinositide 3-kinase; PKC: protein kinase C; HDAC: histone deacetylase; PHD: prolyl hydroxylase; VHL: von Hippel-Lindau; FIH-1: factor inhibiting HIF-1; CBP: CREB binding protein; SDH: succinate dehydrogenase; FH: fumarate hydratase; IDH3: isocitrate dehydrogenase 3; USP20: ubiquitin specific peptidase 20; VDU2: von Hippel-Lindau protein-interacting deubiquitinating enzyme-2; USP8: ubiquitin specific peptidase 8; UCHL1: ubiquitin C-terminal hydrolase L1; WSB1: tryptophan-aspartic acid (WD) repeat and suppressor of cytokines signaling (SOCS) box-containing 1. Black arrows show regulatory steps of HIF-1 activity, and red arrows and blue T bars show positive and negative impacts on them, respectively.

Accumulating evidence has suggested that mechanisms regulating the stability and transactivating activity of HIF-1α protein exhibit the greatest impact on HIF-1 activity [18] (Figure 1, Table 1). Proline residues at positions 402 and 564 (P402 and P564, respectively) of HIF-1α are hydroxylated by prolyl-4-hydroxylase (PHDs) in an oxygen-, Fe^2+^-, and α-ketoglutarate (α-KG)-dependent manner [19,20]. The hydroxylated HIF-1α is then immediately ubiquitinated by E3 ubiquitin ligase containing the von Hippel-Lindau tumor suppressor protein (pVHL) and degraded through the 26S proteasome [21,22,23]. Just like PHDs, factor inhibiting HIF-1 (FIH-1) has also been identified as an oxygen-requiring hydroxylase for HIF-1α [24]. FIH-1 inhibits the transactivation activity, but not stability, of the HIF-1α protein through asparaginyl hydroxylation of HIF-1α at position 803 (N803), thereby suppressing its interaction with the histone acetyltransferase p300/CREB binding protein (CBP) [18,24,25]. Because the oxygen affinity of PHDs is relatively lower than that of FIH-1, PHDs become inactive prior to FIH-1 when the oxygen availability gradually decreases from normoxic to hypoxic conditions [26,27,28]. Therefore, HIF-1α hydroxylated at N803 accumulates under mild hypoxia, after which it acquires full transactivating activity under relatively severe hypoxic conditions due to the inactivation of FIH-1. The stabilized HIF-1α interacts with its binding partner, HIF-1β, and the resultant heterodimer, HIF-1, induces the expression of a series of hypoxia-responsive genes by binding to the hypoxia-responsive enhancer sequence, hypoxia-response element (HRE) [3].

### 2.2. Non-Canonical Mechanisms

HIF-1 is considered a master regulator of cellular adaptive responses to hypoxia because of its hypoxia responsiveness. However, advancements in the field of HIF-1 biology have revealed that HIF-1 functions even under normoxic conditions due to cancer cell-specific gene mutations or an aberrant gene expression profile (Table 1). For example, it has been reported that HIF-1 activity is upregulated through various mechanisms, as follows: Aberrant activation of the phosphoinositide 3-kinase (PI3K)/Akt/protein kinase C (PKC)/histone deacetylase (HDAC) pathway increases the transcription initiation of the *HIF1A* gene [29]. Activation of the PI3K/Akt pathway upregulates the efficiency of the translation initiation of the HIF-1α protein [32]. Deficiency of functional pVHL decreases the ubiquitination and subsequent proteolysis of HIF-1α [21,22,23]. Overexpression of deubiquitinating enzymes, such as ubiquitin C-terminal hydrolase L1 (UCHL1) [41,42,45], ubiquitin specific peptidase 20 (USP20/VDU2) [39], or ubiquitin specific peptidase 8 (USP8) [40] causes deubiquitination and resultant stabilization of HIF-1α. Ubiquitination and subsequent degradation of pVHL triggered by tryptophan-aspartic acid (WD) repeat and suppressor of cytokines signaling (SOCS) box-containing 1 (WSB1) also causes stabilization of the HIF-1α protein [43]. It remains unclear how the accumulated HIF-1α escapes the suppressive effect of FIH-1 and subsequently gains transcription activity under normoxic conditions.

In addition, disorders in the carbohydrate metabolic pathway have also been reported to induce HIF-1 activity of cancer cells even under normoxic conditions. The hydroxylase activity of both PHD and FIH-1 require not only molecular oxygen as a substrate, but also α-KG as a co-factor, as described above. Therefore, a decrease in the intracellular α-KG levels due to overexpression of the α subunit of isocitrate dehydrogenase 3 (IDH3), IDH3α [37], or mutations and resultant amino acid substitutions in succinate dehydrogenase (SDH) or fumarate hydratase (FH) in the TCA cycle of cancer cells results in the activation of HIF-1 by keeping P402, P564, and N803 unhydroxylated, even under normoxic conditions [35,36,37]. Thus, the molecular mechanisms by which HIF-1α accumulates even in the presence of oxygen have been elucidated one after another, making it possible to understand why the HIF-1α protein is detected in the proximal regions of tumor blood vessels in clinical cancer tissues.

## 3. Functions of HIF-1 in the Warburg Effect: Switch from Mitochondrial OXPHOS to Aerobic Glycolysis

### 3.1. Induction of Aerobic Glycolysis

**Figure 2:**
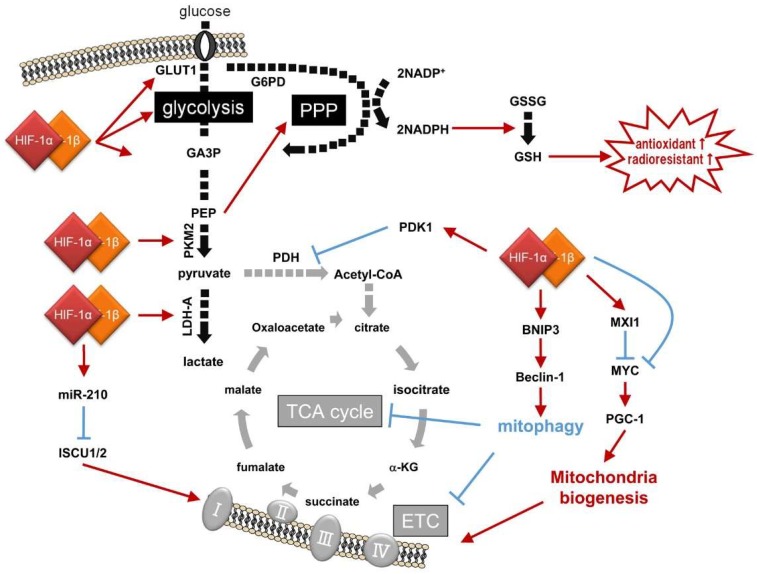
HIF-1-dependent reprogramming of the glucose metabolic pathway, and resultant radioresistance. GA3P: glyceraldehyde-3-phosphate; PEP: phosphoenol pyruvic acid; GLUT1: glucose transporter 1; LDH-A: lactate dehydrogenase-A; GAPDH: glyceraldehyde-3-phosphate dehydrogenase; MCT4: Monocarboxylate transporter 4; PDH: pyruvate dehydrogenase; PDK1: PDH kinase 1; ISCU 1/2: iron-sulfur cluster assembly protein 1/2; MXI1: MAX Interactor 1; PGC-1α: alpha subunit of peroxisome proliferator-activated receptor gamma coactivator 1; BNIP3: B-cell lymphoma 2 (BCL2)-interacting protein 3; PKM2: pyruvate kinase M2; G6PD: glucose-6-phosphate dehydrogenase; GAP: glyceraldehyde-3-phosphate; 1,3-BPG: 1,3-bisphosphoglycerate; acetyl-CoA: acetyl coenzyme A; Bcl-2: B-cell lymphoma 2; G6P: glucose-6-phosphate; PPP: pentose phosphate pathway. Black and gray arrows show active and inactive glucose metabolic pathways, respectively. Red arrows and blue T bars show positive and negative impacts on the pathways, respectively.

Glycolysis is a metabolic pathway that produces two molecules each of pyruvate and ATP from a glucose molecule through sequential and oxygen-independent enzymatic reactions (Figure 2; Table 2). The first step is glucose uptake. Twelve types of glucose transporters (GLUT1-12) function in glucose uptake into human cells. It is widely known that expression of the rate-limiting enzyme for glycolysis, GLUT1, is under the positive regulation of HIF-1 [46]. Genetic alterations in cancer cells, as well as hypoxic stimuli, have been reported to induce GLUT1 expression in a HIF-1-dependent manner, increase cellular glucose uptake, and support the aerobic glycolysis of cancer cells.

In addition to GLUTs, expressions of other glycolytic enzymes have also been demonstrated to be induced by HIF-1, as Iyer et al. first reported in 1998 by culturing a human hepatocellular carcinoma cell line under hypoxic conditions [48]. Pyruvate produced through glycolysis is further metabolized to lactate rather than to acetyl coenzyme A (acetyl-CoA) through lactic acid fermentation mediated by lactate dehydrogenase A (LDH-A) in cancer cells expressing high levels of LDH-A in a HIF-1-dependent manner [47,48]. The lactic acid fermentation plays an indispensable role in maintaining glycolysis because it produces NAD^+^, which is an essential coenzyme for the sixth reaction in glycolysis, the conversion of glyceraldehyde-3-phosphate (GAP) to 1,3-bisphosphoglycerate (1,3-BPG) by glyceraldehyde-3-phosphate dehydrogenase (GAPDH). Monocarboxylate transporter 4 (MCT4), whose expression is also HIF-1-dependent, functions in lactate efflux for intracellular pH homeostasis [49].

### 3.2. Suppression of Mitochondrial Function

Another regulatory component leading to the Warburg effect is suppression of the mitochondrial function, which is accomplished mainly through the following three mechanisms (Figure 2, Table 2).

The first is the inactivation of the TCA cycle due to a decrease in the levels of its initial metabolite, acetyl-CoA. Acetyl-CoA is produced from the end product of glycolysis, pyruvate, through a process called “pyruvate decarboxylation” or “pyruvate oxidation”. The process is mediated by the pyruvate dehydrogenase (PDH) complex, whose first component enzyme is pyruvate dehydrogenase E1α (PDH-E1α) [52,53]. Because PDH-E1α activity is suppressed through its phosphorylation by pyruvate dehydrogenase kinase 1 (PDK1) and HIF-1 is responsible for the expression of PDK1, activation of HIF-1 leads to a decrease in acetyl-CoA levels and inactivation of the TCA cycle [54,55].

The second is through reduction in the levels of proteins associated with mitochondrial functions. The enzyme mediating the isomerization of citrate to isocitrate in the TCA cycle, aconitase, as well as enzymes in mitochondrial complex I, are both important for mitochondrial activity and require the assembly of iron-sulfur clusters mediated by the iron-sulfur cluster assembly protein 1/2 (ISCU1/2) for their function [56]. It has been reported that microRNA-210 (miR-210), whose expression is induced by HIF-1, directly targets ISCU 1/2-encoding mRNAs and suppresses their expression; therefore, activation of HIF-1 results in a decline in the mitochondrial function [57].

The third is through an active decrease in the number of mitochondria by HIF-1-mediated inhibition of mitochondrial biogenesis and induction of mitochondrial autophagy, mitophagy. HIF-1 has been reported to repress the activity of an oncogene, c-Myc, for the suppression of mitochondrial biogenesis through the following two mechanisms. First, HIF-1 directly induces the expression of a negative regulator of c-Myc, MAX Interactor 1 (MXI1), which inhibits the transcriptional activity of c-Myc by competing for MAX, a protein supporting c-Myc [58,59]. Also, HIF-1 functions in the degradation of the c-Myc protein via the proteasome pathway [58]. The number of intracellular mitochondria decreases when c-Myc activity is suppressed, because c-Myc upregulates the expression of a positive transcriptional regulator of mitochondrial biogenesis, the alpha subunit of peroxisome proliferator-activated receptor gamma coactivator 1 (PGC-1α).

As for the active decrease in the number of mitochondria through mitophagy, Beclin-1- and Atg5-dependent mechanisms have been reported to be promoted when HIF-1 induces the expression of a B-cell lymphoma 2 (Bcl-2) family-member protein, BCL2-interacting protein 3 (BNIP3) [60,61]. Beclin-1 protein, which works as the origin of autophagosome formation in autophagy, is usually kept inactive through interaction with Bcl-2 [63]. However, once HIF-1-induced BNIP3 physically interacts with Bcl-2, it promotes the release of Beclin-1 from Bcl-2 and induces mitophagy [60,61]. The BNIP3-dependent reduction in the number of mitochondria has been confirmed to play a very important role in cellular adaptive responses to hypoxia based on the following experimental results: inhibition of either HIF-1α or BNIP3 led to the production of reactive oxygen species (ROS) in mitochondria and eventually induced cell death under hypoxic conditions; however, in the case of mouse embryonic fibroblasts (MEFs) derived from a HIF-1α knockout mouse, cell death was rescued by the forced expression of BNIP3 [60].

## 4. Significance of the HIF-1-Dependent Warburg Effect

Glycolysis is a less efficient pathway compared with mitochondrial OXPHOS in terms of the amount of ATP produced from one molecule of glucose. However, there may be reasons why cancer cells choose such an inefficient glucose metabolic pathway even under normoxic conditions.

### 4.1. Activation of HIF-1 and Angiogenesis by Lactate Uptake

Lactate produced as an end-product of lactic acid fermentation following glycolysis is exported from cells by MCT4 [50,64]. Cancer cells then uptake the lactate using monocarboxylate transporter 1 (MCT1) [65] and convert it to pyruvate by the enzymatic activity of lactate dehydrogenase-B (LDH-B) [66]. An increase in the intracellular levels of pyruvate suppresses the production of α-KG, which stabilizes HIF-1α and activates HIF-1, leading to the induction of VEGF-dependent tumor angiogenesis and acceleration of tumor growth [67].

### 4.2. Effect of HIF-1 on the Activation of the Pentose Phosphate Pathway, Nucleotide Biogenesis, and Antioxidant Potential

Among the glycolytic enzymes whose expression is HIF-1-dependent, pyruvate kinase M2 (PKM2) has been reported to influence the activity of the pentose phosphate pathway [62]. Because the enzymatic activity of PKM2 is weaker than that of other isoforms, the pyruvate kinase (PK) complex, when it contains PKM2 as a component, becomes unable to efficiently convert phosphoenolpyruvate to pyruvate. It has also been reported that the enzymatic activity of PKM2 further decreases when C358 is oxidized by ROS [60]. As a result of the decrease in PK activity and subsequent restriction of glycolytic flux, an intermediary metabolite of glycolysis, glucose-6-phosphate (G6P), is supplied to the pentose phosphate pathway. Because the pathway generates not only various kinds of pentoses, which are used for the biogenesis of nucleotides such as ribonucleotide and deoxyribonucleotide, but also the reduced form of nicotinamide adenine dinucleotide phosphate (NADPH), which is used for the production of the antioxidant, reduced glutathione (GSH), activation of this pathway enhances the antioxidant capacity and radioresistance of cancer cells.

## 5. The Warburg Effect and Radioresistance of Cancer Cells Mediated by Novel Activators of HIF-1

Radioresistance caused by induction of the Warburg effect by HIF-1 results in cancer cells that are difficult to treat and that may lead to tumor recurrence. As such, activators of HIF-1 represent a promising group of targets that may lead to the development of novel therapies.

### 5.1. Effects of microRNAs on the Regulation of HIF-1

Interestingly, there has been emerging evidence that there are numerous microRNAs that participate in HIF-1-mediated regulation of the Warburg effect. miR-31-5p, a microRNA that is upregulated in lung adenocarcinoma and is implicated to play roles in oncogenesis, has been found to enhance the Warburg effect and promote cell proliferation through the canonical HIF-1 regulatory pathway [68]. It has been found to do so by inhibiting FIH-1, leading to a rise in HIF-1 transactivation activity. This results in the appearance of hallmarks of aerobic glycolysis, such as elevated glucose uptake and lactate levels, as well as an increase in OXPHOS-independent ATP production [68]. Inhibition of miR-31-5p and FIH-1 overexpression were able to attenuate these effects [68]. Similarly, miR-150, another microRNA that is aberrantly expressed in cancers, has been found to target and inhibit pVHL in glioma cells [69]. As pVHL is the E3 ligase responsible for HIF-1 degradation, miR-150 stabilizes the HIF-1 protein [69]. Overexpression of miR-150 was found to increase glucose uptake, lactate production, and cell proliferation [69]. These studies have shown that these microRNAs play a role in regulating the HIF-1-dependent induction of the Warburg effect; however, they have not yet evaluated if and how these microRNAs contribute to the resulting increase in antioxidants and radioresistance in cancer cells. Further study in this area may lead to clues on how to radiosensitize cancer cells and increase treatment options.

### 5.2. Effects of Novel Factor UCHL1 on the Regulation of HIF-1

Our own genetic screening experiments for exploring novel activators of HIF-1 [30,37,41] have led us to identify a unique factor that induces cancer cell radioresistance through reprogramming of the metabolic pathway. We first constructed a plasmid that expressed an enzyme for blasticidin S-resistance, blasticidin-S deaminase (BSD), under the control of the HIF-1-dependent 5HRE promoter (5HREp), and established a stable transfectant with it. The stable cells were then introduced with a human cDNA library and cultured in the presence of blasticidin-S under normoxic conditions with the expectation that some of the cDNA would lead to the survival of colonies through the activation of HIF-1 and resultant expression of BSD. We successfully acquired several surviving colonies, analyzed cDNA responsible for the survival, and eventually identified UCHL1 [41], IDH3α [37], and lymphocyte antigen 6 locus E (LY6E) [30] as novel activators of HIF-1.

In vitro experiments revealed that UCHL1 stabilizes the HIF-1α protein with its deubiquitination activity [41,42]. When we quantitatively analyzed intermediary metabolites of the glucose metabolic pathway after overexpressing UCHL1, we found that these genes induced reprogramming of the glucose metabolic pathway from mitochondrial OXPHOS to glycolysis [42]. The accelerated glycolysis was confirmed to be accompanied by activation of the pentose phosphate pathway, leading to the production of both NADPH and an antioxidant, reduced glutathione (glutathione-SH: GSH) [42]. In vitro colony formation assays demonstrated that the forced expression of UCHL1 significantly increased GSH levels and subsequently induced the radioresistance of cancer cells [42]. UCHL1-dependent radioresistance was abrogated when the HIF-1α gene was silenced with siRNA or when intracellular GSH levels were pharmacologically decreased with an inhibitor of the rate-limiting enzyme of the pentose phosphate pathway, glucose-6-phosphate dehydrogenase (G6PD) [42]. Finally, clinical research confirmed that the expression levels of UCHL1 were positively correlated with that of HIF-1α in malignant solid tumors and the poor prognosis of breast and lung cancer patients [41].

These results indicate that the UCHL1-mediated activation of HIF-1 leads to the radioresistance of cancer cells through inducing the Warburg effect, and suggest the possibility that UCHL1, as well as HIF-1, could be exploited as rational targets for enhancing the effects of radiotherapy and markers for predicting the effects.

## 6. Conclusions and Future Direction

In this review, we focused on the reprogramming of the glucose metabolic pathway in cancer cells, especially on the Warburg effect, and summarized the HIF-1-dependent mechanism and function behind it. Accumulated knowledge has revealed that cancer cells hijack the sophisticated systems in tissue cells to acquire antioxidant properties through reprogramming the glucose metabolic pathway. Elucidating the mechanistic and functional interplays between HIF-1 and factors functioning in the regulation of glucose metabolism in cells is expected to deepen our understanding of the complex features of cancer cells.

Although we highlighted the importance of HIF-1 in the induction of the Warburg effect, we should not ignore the involvement and influences of other factors. For example, Dang suggested that because oncogenic c-Myc and hypoxia-inducible factor (HIF) collaborate to increase the uptake of glucose and its conversion to lactate and enhance the cancer cell’s metabolic needs, their common downstream target genes, such as *PDK1* and *LDHA*, can be utilized as attractive therapeutic targets [70]. Moreover, the Warburg effect-like glucose metabolism was reported in a HIF-1α-deficient clear cell renal cell carcinoma cell line [71].

Each cancer cell exhibits different metabolic properties, even in identical tumor tissues; for example, some cancer cells prefer glycolysis, but others prefer OXPHOS; this preference is assumed to be caused by things like genetic heterogeneity within a tumor, the influences of oxygen microenvironments, and/or by interplay with surrounding non-cancer cells. Research on the metabolic pathway focusing on the tumor’s microenvironment and on the interplay between cancer and non-cancer cells in vivo is needed to deepen our understanding of the nature of cancers and develop novel strategies for cancer therapy.

## Figures and Tables

**Table 1:** List of positive and negative regulators of HIF-1.

Gene Products	Mechanism Regulating HIF-1 Activity	References
Transcription initiation of the *HIF1A* gene		
PI3K/Akt/PKC/HDAC pathway	Upregulating transcription initiation in case that mitochondrial ND6 gene harbors G13997A mutation	[29]
LY6E	Activating the PI3K/Akt pathway through the decrease in PTEN expression	[30]
Translation initiation of the *HIF1A* gene		
PI3K/Akt pathway	Upregulating both cap-dependent and IRES-dependent translation initiation	[31,32,33]
Stability of the HIF-1α protein by modulating its prolyl hydroxylation status		
PHD1, 2, 3	hydroxylating P402 and P564 of HIF-1α for ubiquitination	[19,20,34]
LOF mutant of SDH	Inactivation of PHDs and FIH-1 through the “product inhibition” due to abnormal accumulation of succinate	[35]
LOF mutant of FH	Inactivation of PHDs and FIH-1 through the “product inhibition” due to abnormal accumulation of fumarate	[36]
IDH3	Inactivating PHDs through the decrease in 2OG levels, when overexpressed aberrantly.	[37]
Stability of the HIF-1α protein by modulating its ubiquitination status		
pVHL	Ubiquitinating HIF-1α for its proteasomal degradation	[21,22,38]
USP20/VDU	Deubiquitinating HIF-1α for its stabilization	[39]
USP8	Deubiquitinating HIF-1α for its stabilization	[40]
UCHL1WSB1	Deubiquitinating HIF-1α for its stabilization Ubiquitination of pVHL	[41,42] [43]
Transactivation activity of the HIF-1α protein		
FIH-1	Hydroxylating N803 of HIF-1α to inhibit the interaction of HIF-1α with p300/CBP	[24,44]
IDH3	Inactivating FIH-1 through the decrease in 2OG levels, when overexpressed aberrantly.	[37]
p300/CBP	Interacting with HIF-1α and functioning as a co-activator with their histone acetyltransferase activity	[18,24,25]

**Table 2:** List of genes influencing the Warburg effect.

Gene Products	Function	Toward the Warburg Effect	References
GLUT1	Increase in glucose uptake to promote glycolysis	Positive	[46]
LDH-A	Hydrogenation of pyruvate to lactate in lactic acid fermentation	Positive	[47,48]
GAPDH	Catalyzing dehydrogenation of GAP to 1,3-BPG in glycolysis	Positive	[47,48]
MCT4	Efflux of lactate	Positive	[49,50,51]
PDH	Catalyzing oxidative decarboxylation of pyruvate to acetyl-CoA	Negative	[52,53]
PDK1	Phosphorylating PDH for its inhibition	Positive	[54,55]
ISCU 1/2	Facilitating the assembly of aconitase and enzymes of the mitochondrial complex I for their function	Negative	[56,57]
MXI1	Inhibiting c-Myc transcription activity by competing for MAX, a supporting protein to c-Myc	Positive	[58,59]
PGC-1α	Inducing the expression of transcription regulators for mitochondrial biogenesis	Negative	[58]
BNIP3	Interacting with Bcl-2 to dissociate Beclin-1 from Bcl-2 for mitophagy	Positive	[60,61]
PKM2	Regulating glycolytic flux and supply G6P to the PPP	No influence	[62]
G6PD	Catalyzing the conversion of G6P to 6-phospho-glucono-1,5-lactone, and functioning as a rate-limiting enzyme for the PPP	No influence	[41,42,45]

GLUT1: glucose transporter 1; LDH-A: lactate dehydrogenase-A; GAPDH: glyceraldehyde-3-phosphate dehydrogenase; MCT4: monocarboxylate transporter 4; PDH: pyruvate dehydrogenase; PDK1: PDH kinase 1; ISCU 1/2: iron-sulfur cluster assembly protein 1/2; MXI1: MAX Interactor 1; MAX: c-Myc associated factor X; PGC-1α: alpha subunit of peroxisome proliferator-activated receptor gamma coactivator 1; BNIP3: BCL2-interacting protein 3; PKM2: pyruvate kinase M2; G6PD: glucose-6-phosphate dehydrogenase; GAP: glyceraldehyde-3-phosphate; 1,3-BPG: 1,3-bisphosphoglycerate; acetyl-CoA: acetyl coenzyme A; Bcl-2: B-cell lymphoma 2; G6P: glucose-6-phosphate; PPP: pentose phosphate pathway.
